# DeepMatch: Toward Lightweight in Point Cloud Registration

**DOI:** 10.3389/fnbot.2022.891158

**Published:** 2022-07-18

**Authors:** Lizhe Qi, Fuwang Wu, Zuhao Ge, Yuquan Sun

**Affiliations:** ^1^Intelligent Industrial Robot and Intelligent Manufacturing Laboratory, Ministry of Education's Engineering Research Center of AI and Robotics, Academy for Engineering and Technology, Fudan University, Shanghai, China; ^2^Intelligent Industrial Robot and Intelligent Manufacturing Laboratory, Shanghai Engineering Research Center of AI and Robotics, Academy for Engineering and Technology, Fudan University, Shanghai, China

**Keywords:** 3D vision, point cloud registration, algorithms, datasets, transformation

## Abstract

From source to target, point cloud registration solves for a rigid body transformation that aligns the two point clouds. IterativeClosest Point (ICP) and other traditional algorithms require a long registration time and are prone to fall into local optima. Learning-based algorithms such as Deep ClosestPoint (DCP) perform better than those traditional algorithms and escape from local optimality. However, they are still not perfectly robust and rely on the complex model design due to the extracted local features are susceptible to noise. In this study, we propose a lightweight point cloud registration algorithm, DeepMatch. DeepMatch extracts a point feature for each point, which is a spatial structure composed of each point itself, the center point of the point cloud, and the farthest point of each point. Because of the superiority of this per-point feature, the computing resources and time required by DeepMatch to complete the training are less than one-tenth of other learning-based algorithms with similar performance. In addition, experiments show that our algorithm achieves state-of-the-art (SOTA) performance on both clean, with Gaussian noise and unseen category datasets. Among them, on the unseen categories, compared to the previous best learning-based point cloud registration algorithms, the registration error of DeepMatch is reduced by two orders of magnitude, achieving the same performance as on the categories seen in training, which proves DeepMatch is generalizable in point cloud registration tasks. Finally, only our DeepMatch completes 100% recall on all three test sets.

## Introduction

With the development of modern hardware, such as depth cameras and lidar, many tasks have been extended to three-dimensional point clouds. Important fields, such as robotics (Deschaud, 2018; Han et al., 2019), autonomous driving (Wan et al., 2018; Lu et al., 2019; Li et al., 2020a), and medical imaging (Yoo et al., 2020), all rely on point cloud registration. The goal of point cloud registration is to calculate the homogeneous transformation matrix, namely, rotation matrix R and translation vector t, according to the original point cloud and the target point cloud, so that the original point cloud can be as close to the target point cloud as possible after transformation.

In this study, the global optimal alignment can be solved by singular value decomposition (SVD) given the exact point correspondence. In addition, it becomes easier to calculate matches if the global alignment information was known. Many algorithms iterate between them because these two steps depend on each other. However, the resulting iterative optimization algorithm tends to be locally optimal. As the most classical point cloud registration algorithm, the iterative closest point (ICP) algorithm (Besl and McKay, 1992) often stagnates at suboptimal local minima because of the non-convexity of the problem. A series of improved methods (Rusinkiewicz and Levoy, 2001; Fitzgibbon, 2003), such as GO-ICP (Yang et al., 2015), have tried to alleviate this problem based on branch-and-bound (BnB), but still do not always provide acceptable output (Wang and Solomon, 2019a; Choy et al., 2020), and these algorithms are computed slower than ICP because of the time-consuming BnB (Aoki et al., 2019; Wang and Solomon, 2019a; Fu et al., 2021). Deep Closest Point (DCP) (Wang and Solomon, 2019a), an improved algorithm of ICP, makes pioneering use of the neural network to extract per-point features and calculates the point-to-point correspondence through the similarity of the per-point feature of two-point clouds. DCP and its improved algorithms (Fu et al., 2021) are still not perfectly robust and rely on complex model design because the extracted local features are susceptible to noise.

In this study, we reviewed the limitations of the DCP and its improved algorithm and proposed the DeepMatch algorithm, which extracts a new per-point feature for the point cloud. As proved by experiments, point cloud registration can be completed more efficiently and accurately on this per-point feature. Our model simply consists of three parts, as follows: (1) Point Structure Extractor to extract a per-point structure, (2) the convolution part of the bonnet, which is a very simple 4-layered convolution, and (3) the differentiable singular value decomposition part, predicting rigid body transformation. This means that we omit the redundant pointer part of DCP, which occupies the most computing resources and does not help much to improve the effect, and DeepMatch does not need to use more complex feature extractors like other algorithms. We also train and test on ModelNet40, and the accuracy of registration exceeds DCP and its improved algorithm, leading to the point cloud registration algorithm. At the same time, the computing resources and training time we need are much less than existing registration algorithms.

Our main contributions to this study include the following two aspects. First, we propose to use DeepMatch to extract a per-point feature from a new per-point structure. Based on this feature, our DeepMatch achieves state-of-the-art (SOTA) performance on clean, noisy, and unseen categories datasets with at most one-tenth of the computing resources (GPU memory) and computing time of other similarly performing learning-based algorithms. The robustness of DeepMatch is also excellent, with a recall rate of 100% in all three datasets. Second, we proved that learning-based registration depends on the point cloud size. By scaling the point cloud before registration, compared with the best learning-based method before, the accuracy of our DeepMatch on the unseen point cloud was improved by two orders of magnitude.

## Related Works

### Traditional Ways of Point Cloud Registration

One of the most important ways of traditional the point cloud registration algorithm is to obtain the final rigid body transformation using an iterative method, among which the most classic is the ICP algorithm. The ICP algorithm has very high registration accuracy when it can complete the registration, but ICP is a non-convex problem, so it is very easy to fall into the local optimal solution when the source point cloud and the target point cloud are in a bad initial position. The improved normal iterative closest point (NICP) algorithm (Jia et al., 2016) improves the registration speed and accuracy by eliminating the wrong corresponding points through the local features of the points, and the point-to-line ICP algorithm(Censi, 2008) and point-to-plane ICP algorithm (Low, 2004) change the correspondence between points and points to the correspondence between points and lines, and points and planes, respectively, but they still have not solved the problem of poor ICP robustness. The Go-ICP, GOGMA (Campbell and Petersson, 2016), and other algorithms (Yang et al., 2015; Campbell et al., 2019) alleviate this problem with the global optimization algorithm based on BnB, but these algorithms cannot guarantee the completion of registration, and another defect is their slow computing speed.

PFH (Rusu et al., 2008) and its improved algorithm FPFH (Rusu et al., 2009), extract the features of the key points in the point cloud through calculation and build feature descriptors for them, and these feature descriptors are matched to build the potential correspondence. After that, PFH and FPFH used the RANSAC algorithm to complete the final registration. Although these two algorithms based on feature descriptors have good robustness, it is time-consuming to calculate the feature descriptors, and the accuracy of these algorithms is not high.

The fast global registration (FGR) algorithm (Zhou et al., 2016) achieves state-of-the-art performance through hierarchical non-convex strategy convex optimization based on the corresponding objective function and based on the corresponding point cloud registration. However, the correspondence-based approach is sensitive to point clouds of repetitive structures because a large proportion of the potential correspondences in these scenarios are incorrect.

### Learning-Based Registration

One of the earliest algorithms to use deep learning to complete point cloud registration is 3DMatch (Zeng et al., 2017), which, like FPFH, builds 3D local feature descriptors by extracting key points. 3DMatch uses the volumetric grid of truncated distance function to represent the original point cloud in a structured way and uses twin neural networks for training. 3DMatch and its improved algorithms (Gojcic et al., 2019) are not end-to-end, and the calculation of performance better than 3D local feature descriptors is limited, which requires a long registration time.

PointNetLK (Aoki et al., 2019) is the first learning-based end-to-end point cloud registration algorithm that calculates global feature descriptors for two-point clouds through PointNet (Qi et al., 2017) and minimizes the distance between global descriptors using an iterative approach similar to the Lucas-Kanade algorithm (Lucas and Kanade, 1981; Baker and Matthews, 2004). PCRNet (Sarode et al., 2019) replaces the Lucas-Kanade algorithm in PointNetLK with a deep neural network. But they can still fall into the local optimal solution, and they do not take advantage of local features.

Deep ClosestPoint proposed to use the deep learning method to obtain a per-point feature to improve ICP. In DCP, the structure composed of each point and its nearest k points is the feature to be extracted, that is, they use DGCNN (Wang et al., 2019) to extract the edge graph composed of each point and its neighborhood. Based on DCP, PRNet (Wang and Solomon, 2019b) proposes a key-point detector and uses the key-point to key-point relationship to solve the partial-to-partial-point cloud registration in a self-supervised way. RGM (Fu et al., 2021) proposed depth map matching, which used the features of other nodes and the structure information of graphs to establish the corresponding relationship, and introduced the AIS module to establish the reliable corresponding relationship between the nodes of two given graphs. Since the nearest point is easily affected by noise, the feature they used is only proved to be very effective in point cloud registration without noise or with a small amount of noise, which means that the source point cloud and the target point cloud need to have the point structure extremely high similarity, even to be the same on some objects, and because of its sparseness, point clouds may represent the same object with completely different point structures. In addition, the feature extraction of this edge graph depends on the feature extraction capability of the complex network model structure. CorsNet (Yuan et al., 2020) concatenates the local features with the global features and regresses correspondences between point clouds, and more useful information is integrated than the conventional approaches. Algorithms such as DeepGMR (Li et al., 2020b) and IDAM (Wu et al., 2015) have proposed some new methods to solve the problems of DCP and PointNetLK, but the accuracy of these algorithms is insufficient.

## Problem Statement

In this section, we will formulate the 3D point cloud registration problem and our DeepMatch to facilitate your understanding of the study.

Given the source point cloud P = {p_i_ ϵ R^3^|i = 1, 2, ..., M} and the target point cloud Q = {q_j_ ϵ R^3^|j = 1, 2, ..., N}, from source to target, the task of point cloud registration is to solve a rigid body transformation {R, t}, which makes P coincide with Q as much as possible. R ϵ SO(3) is a rotation matrix, and t ϵ R^3^ is a translation vector. It is not required that the source point cloud and the target point cloud have the same number of points, that is to say, M may not be equal to N.

For any point p_i_ in the source point cloud, assuming that the point q_i_ in the target point cloud is the corresponding point of p_i_, the goal of the registration algorithm is to obtain the corresponding pose by minimizing the distance between the corresponding point pairs:


(1) $$E\left(R,t\right)=\underset{R\epsilon SO(3),t\epsilon R^{3}}{\mathrm{argmin}}\sum_{i=1}^{n}w_{i}\;\|(Rp_{i}\;+t)-q_{i}\|$$

where *w*_*i*_ represents the weight of each pair of corresponding points. Here, we assume that the point-to-point mapping relationship is known and expressed by the function *m*, that is, *q*_*i*_ = *m*(*p*_*i*_), and the objective function *E*(*R, t*) should be modified to:


(2) $$E\left(R,t\right)=\underset{R\epsilon SO(3),t\epsilon R^{3}}{\mathrm{argmin}}\sum_{i=1}^{n}w_{i}\;\|(Rp_{i}\;+t)-m(p_{i})\|$$

The solutions of Equation (2) depend on the solution of the mapping function m. In DeepMatch, we used deep learning to estimate the mapping function m, that is, suppose F_P_ and F_Q_ are the feature embeddings we extracted, the mapping function m can be calculated according to the similarity of F_P_ and F_Q_ as follows:


(3) $$\begin{array}{r} m\left(p_{i}\right)=\sum_{j=1}^{N}\sigma_{ij}q_{j} \end{array}$$

(4) $$\begin{array}{r} \sigma =\;softmax\left(F_{P}F_{Q}^{T}\right) \end{array}$$

The mapped *m*(*p*_*i*_) may not be a real point in the point cloud Q, but the point cloud composed of all *m*(*p*_*i*_) approximately expresses the point cloud Q.

From the above problem statement, we can know that the accuracy of the mapping function m depends on the accuracy of the feature embedding. We will introduce how DeepMatch calculates a more accurate m based on features in the next part.

## Methods

In this section, we will introduce our model as shown in **Figure 2** and describe the driving forces for the improvements made, which explains the simplicity and effectiveness of our model.

### Per-Point Feature

After establishing the above problem statement, it can be easily concluded that the key task is to obtain more accurate point features in the problem of point cloud registration using the deep learning one-shot process.

The edge graph formed by the nearest point is proposed because the coordinates of a single point in the point cloud are obviously meaningless. However, compared with the information of the nearest point, for each point in the point cloud, the position relative to the central point is more effective, as proved by the experiment, which is why we decided to consider the coordinates of the central point when obtaining the features of each point. Our specific approach is to decentralize each point.


(5) $$\begin{array}{r} \bar{p}=\frac{\sum_{i=1}^{n}w_{i}p_{i}}{\sum_{i=1}^{n}w_{i}},\bar{q}=\frac{\sum_{j=1}^{n}w_{j}p_{j}}{\sum_{j=1}^{n}w_{j}} \end{array}$$

(6) $$\begin{array}{r} p_{i}=p_{i}-\bar{p},q_{i}=q_{i}-\bar{q},i=1,2,\ldots ,n \end{array}$$

**Figure 1:**
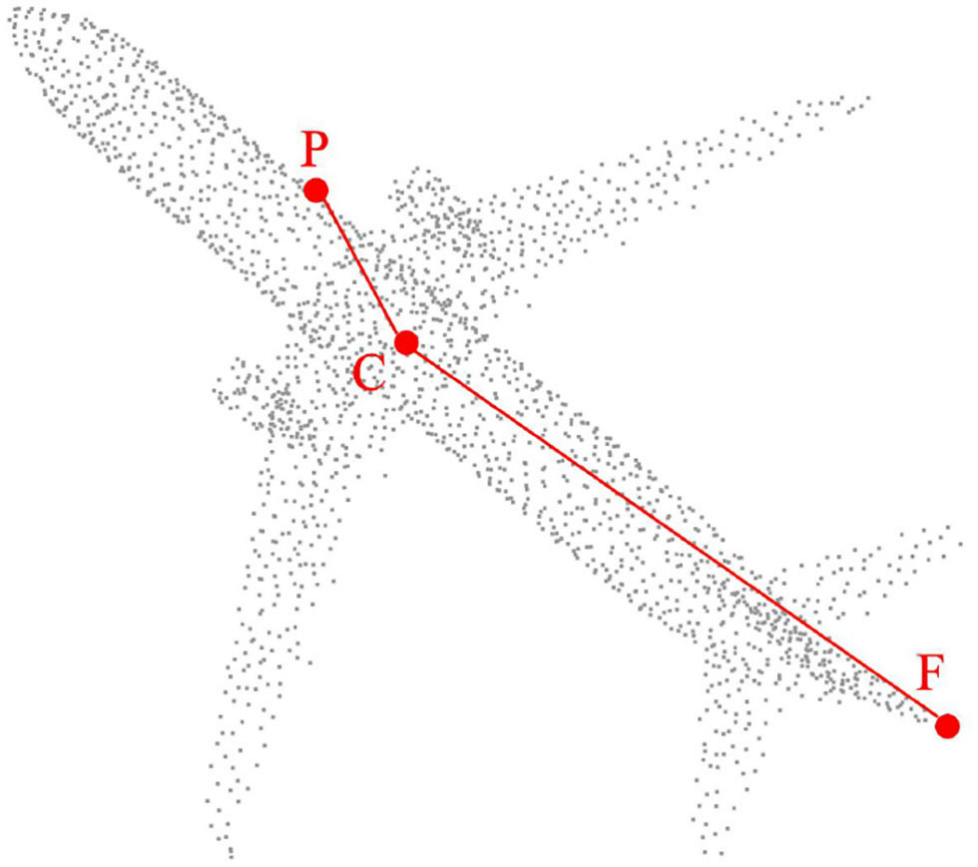
For any point P, DeepMatch extracts a structure composed of point P itself, central point C, and the farthest point F of point P.

In this way, the central point of the point cloud is implied in the coordinate origin O. With just decentralization, we still cannot completely distinguish each point. The feature using the nearest point is very weak in robustness because it is very susceptible to noise. Specifically, the offset distance generated by noise is very large relative to the distance of the nearest point pair. Therefore, we naturally consider the furthest point because the distance offset generated by the noise is almost negligible relative to the furthest point pair. Thus, for any point *p*_*i*_, the structural feature of *p*_*i*_*Of*_*i*_ is formed, where *f*_*i*_ is the farthest point of *p*_*i*_. As shown in Figure 1, our experiments prove the accuracy and robustness of this feature in point cloud registration.

### DeepMatch Network Architecture

**Figure 2:**
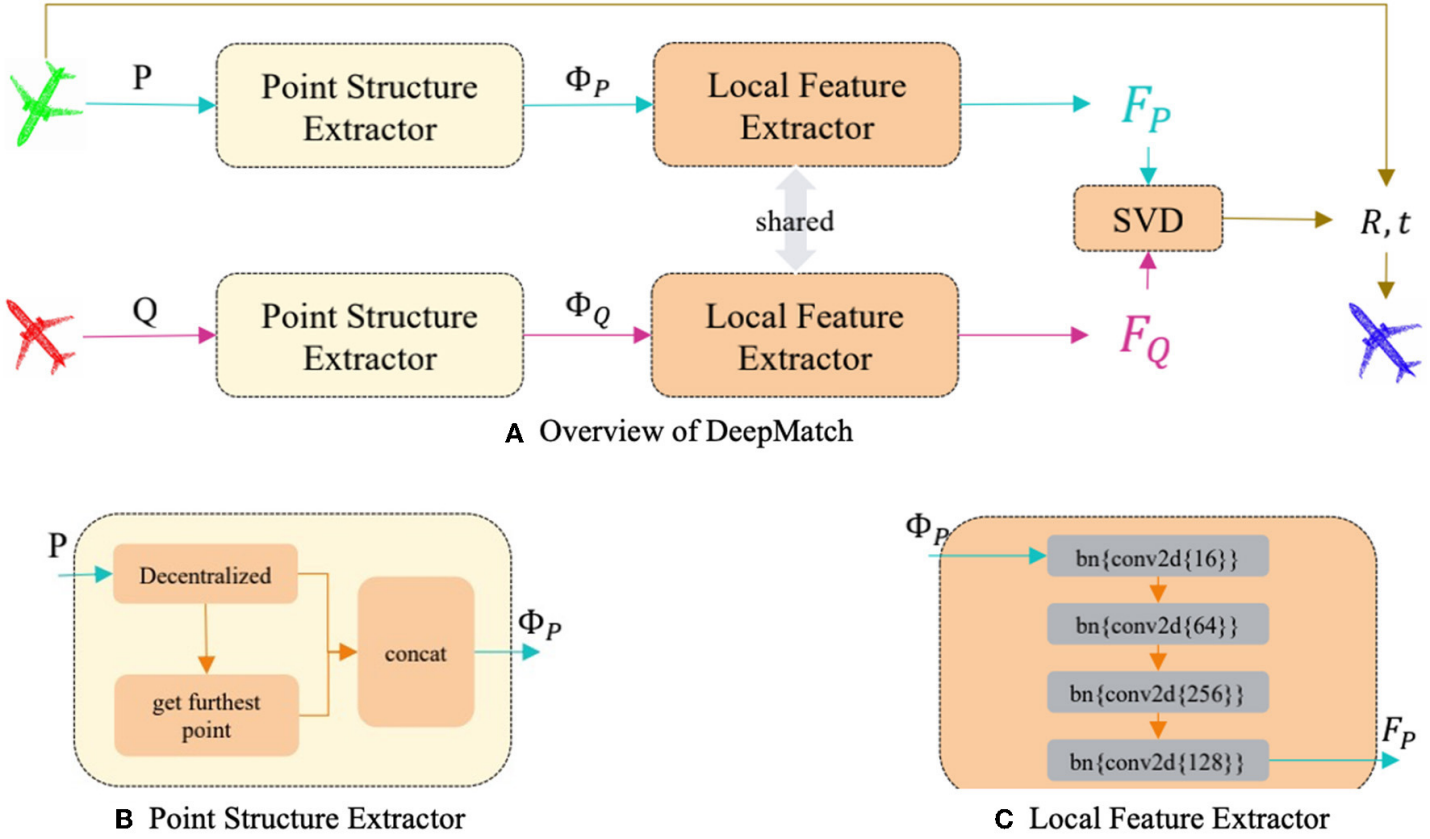
The pipeline of our DeepMatch **(A)** simply consists of Point Structure Extractor, Local Feature Extractor, and a differentiable SVD part, **(B,C)** respectively, show the details of Point Structure Extractor and Local Feature Extractor.

Usually, we used a very complex network structure to complete feature embedding in deep learning; however, our driving force is to improve the effectiveness of features and reduce the complexity of the model, so we tried to use the simplest backbone network possible. After experimental comparison, a four-layered convolutional neural network as shown in Figure 2 can achieve the best performance, the numbers of filters in each layer are (64; 256; 128). Our DeepMatch does not need various models such as transformers that other algorithms rely on to improve feature extraction capabilities. As stated in the problem statement, after obtaining the per-point feature and calculating the corresponding points of two-point clouds through feature similarity, the Singular Value.

The decomposition method should be used to solve the final rigid transformation. In order to make the singular value decomposition process differentiable and thus backpropagated, the covariance matrix of the singular value decomposition is defined as follows:


(7) $$\begin{array}{r} S={P\left(\sigma Q\right)}^{T} \end{array}$$

where σ is defined in Equation (4). The covariance matrix is decomposed into:


(8) $$\begin{array}{r} S=\;U\Sigma V^{T} \end{array}$$

Then the rotation matrix *R* and the translation vector t can be calculated as follows:


(9) $$\begin{array}{r} R=VU^{T},t=\bar{q}-R\bar{p} \end{array}$$

This SVD process is a one-shot process in the deep learning method.

### Scaling Method

We used the following simple mean square error loss:


(10) $$\begin{array}{r} L=||R^{T}R^{g}-\;I||\;+||t-\;t^{g}||\;+{\lambda ||\;\theta ||\;}^{2} \end{array}$$

where R and t are the rotation matrix and translation vector obtained by solving, and R^g^ and t^g^ are the corresponding ground truth. The third term is a regular term, which is used to reduce the complexity of the network. This simple loss is very effective because it is close to the target of the output end.

### Loss Function

On the types of objects that have not been seen, the existing point cloud registration algorithms cannot achieve extremely high accuracy. In the case of the recent RGM, the average error of the rotation matrix is reduced to 1.5457°, but this is obviously not what we want as the result.

After analyzing the reasons for this situation, we conclude as follows. In addition to the poor robustness of relying on the nearest point, the current backbone network mainly extracts distance information for point features, while the size of any two point clouds is obviously different. However, since deep learning can only process quantitative point input, the distance between a point and its closest point in different point clouds may differ by several orders of magnitude.

Therefore, our DeepMatch scales the point cloud before input to the backbone network, so that the size of each input point cloud is about the same. This trick has been proven to solve the problem.

## Experiments

### Per-Point Feature Dataset

Our experiment is based on the ModelNet40 (Choy et al., 2020) dataset, a public dataset containing 12,311 Meshed CAD Models with 40 types of objects. We randomly sampled 1,024 points on the surface of these CAD models as the source point clouds, just as in the experiments of the algorithms such as PointNet and DCP. The rigid transformation [*R*^*g*^, *t*^*g*^] is also generated randomly for each point cloud to obtain the target point clouds, where *R*^*g*^ and *t*^*g*^ are the ground truth of the experimental rotation matrix and translation vector. Similar to DCP and other algorithms, the rotation angle and translation distance of all target point clouds relative to the corresponding source point clouds on each coordinate axis are within the range of [0, 45 m] and [−0.5, 0.5 m], respectively.

### Metrics of the Results

In this study, we used three metrics to measure the performance of each algorithm. The first two are to evaluate the mean absolute error (MAE) between the estimation of the model and the groundtruth of *R* in the rotation axis *XYZ* and t in the direction *XYZ*:


(11) $$\begin{array}{r} MAE\left(R\right)=\frac{\sum_{k=1}^{K}||R_{k}-R_{k}^{g}||}{K} \end{array}$$

(12) $$\begin{array}{r} MAE\left(t\right)=\frac{\sum_{k=1}^{K}||t_{k}-t_{k}^{g}\;||}{K} \end{array}$$

where *K* is the number of point cloud pairs in the test and [$R_{k}^{g}$, $t_{k}^{g}$] denote the ground truth transformation of *k*-th point-cloud pairs. We abandon the mean isotropic errors (MIE) metric introduced in RPM-Net and the clip chamfer distance (CCD) metric introduced in RGM because we believe that these three metrics can clearly measure the accuracy and robustness of each registration algorithm and the redundant indicators will only make the comparison results readable worse. The third metric we used is the recall rate. A recall is considered successful if MAE(R) <1.0° and MAE(*t*) < 0.1 m can be achieved. We measure the number of successful recalls against the total number of tests to get the recall rate.

We also used three metrics to measure the computing resource consumption of each algorithm. The first two are the GPU memory and time required to train these learning-based algorithms, which measure the complexity of each algorithm, and this comparison was done on a workstation with two NVIDIA Quadro P6000 graphics cards. The last is the average time taken to complete a single registration, and this comparison was made on an M1 MacBook pro.

In the traditional point cloud registration algorithm, we selected the most classic ICP algorithm and the better FGR algorithm to compare with us, and the implementation of ICP and FGR is based on the open3d library, and ICP uses an identity matrix as initialization. In addition, we selected four learning-based registration algorithms, namely, RPMNet, IDAM, DeepGMR, and RGM, where RGM represents the latest and most effective registration algorithm before. The latest algorithms have proven that they are superior to earlier learning-based registration algorithms such as DCP and PointNetLK, so these algorithms are not compared in this study. We retrained and tested all learning-based algorithms according to the parameters given in their studies. Since our performance test results are basically the same as those in RGM, we decided to use the performance data given in RGM. All comparison results are displayed in tables, and the best results are marked in bold font.

### Clean Point Cloud

In comparison to clean point clouds, 40 categories of 12,311 point clouds are divided into the training set and test set, among which the training set contains 9,843 point clouds and the corresponding test set has 2,468 point clouds. Both the training set and the test set cover all 40 categories.

**Figure 3:**
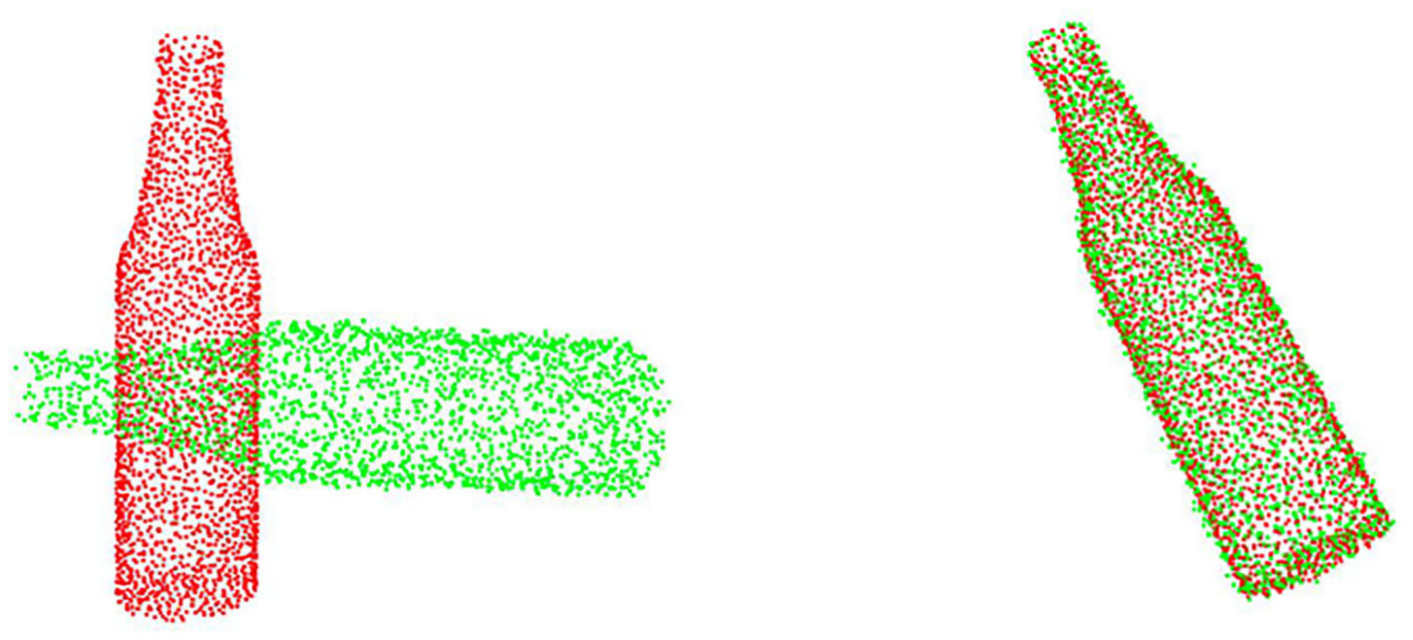
DeepMatch completes the registration under inputs with Gaussian noise.

We trained and tested all of the models mentioned in the previous section, and the results are shown in Table 1. As shown in Figure 3, even though RGM has done a great job in clean point cloud data, we still beat it in MAE(R). Only our DeepMatch had a 100% recall rate. The accuracy of ICP in this test is very poor because its recall rate is very low, which also verifies its shortcomings in poor robustness.

**Table 1:** Performance on clean point clouds.

**Method**	**MAE(R)**	**MAE(t)**	**Recall**
ICP	6.4467	0.05446	74.19%
FGR	0.0099	0.00010	99.96%
RPM-Net	0.2464	0.00112	98.14%
IDAM	1.3536	0.02605	75.81%
DeepGMR	0.0156	0.00002	**100.00%**
RGM	0.0096	<0.00001	**100.00%**
DeepMatch	**0.0095**	**<0.00001**	**100.00%**

### Point Cloud With Gaussian Noise

In the experiment on the point cloud with Gaussian noise, the division of the test set and training set is the same as in the experiment on the clean point cloud. Gaussian noise is added to each point on all point clouds along each of its axes, and this Gaussian noise with a mean of 0 and a variance of 0.01 but the translation in each direction is truncated within the range of [−0.05, 0.05 m]. Obviously, the results of this experiment can intuitively reflect the robustness of the model, and the point cloud data in the actual registration environment are basically with Gaussian noise, so the results of this experiment are more convincing than the experiment on clean point clouds.

Our test results (shown in Table 2) show that our DeepMatch achieves basically the same effect on the point cloud with Gaussian noise as on the clean point cloud, proving that our DeepMatch has extremely high robustness. By comparison, our accuracy is an order of magnitude better than.

**Table 2:** Performance on point clouds with Gaussian noise.

**Method**	**MAE(R)**	**MAE(t)**	**Recall**
ICP	6.5030	0.04944	77.59%
FGR	10.0079	0.07080	30.75%
RPM-Net IDAM	0.5773 3.4916	0.00532 0.02915	96.68% 49.59%
DeepGMR RGM	2.2736 0.1496	0.01498 0.00141	56.52% 99.51%
DeepMatch	**0.0098**	**<0.00001**	**100.00%**

### Performance in Unseen Categories

The purpose of the test on the unseen categories is to prove the learning ability of the model for the point cloud registration task. Because neither ICP nor FGR is a learning-based algorithm, they are not included in this comparison. In this experiment, different from the partitioning method of the test set and training set, we divided the 40 categories into half and half, with the first half for training and the second half for testing. In addition, the point clouds here were all clean point clouds without Gaussian noise.

**Figure 4:**
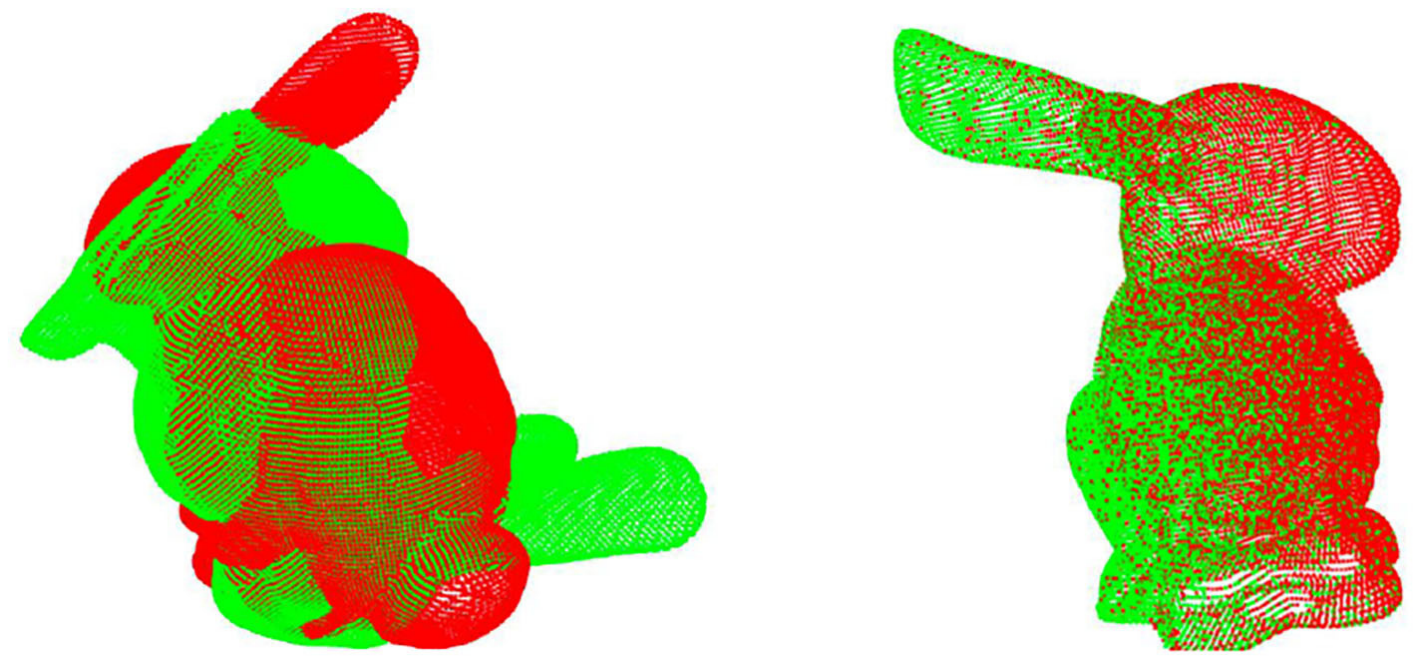
DeepMatch completes the registration on unseen categories.

As shown in Table 3, our performance is far superior to other algorithms on unseen categories, and the accuracy is still consistent with that of the seen clean point cloud dataset. This proves our previous analysis of the impact of point cloud size on registration. The inability to achieve a high recall rate makes the equalization error of other algorithms very high. We also tested on some objects not seen in ModelNet40, such as the Stanford rabbit, as shown in Figure 4, and only our DeepMatch can achieve highprecision registration.

**Table 3:** The performance of different methods on unseen categories of point clouds.

**Method**	**MAE(R)**	**MAE(t)**	**Recall**
RPM-Net	1.9826	0.02276	75.59%
IDAM	19.3249	0.20729	0.95%
DeepGMR RGM	71.0677 1.5457	0.44632 0.01418	0.24% 84.28%
DeepMatch	**0.0107**	**<0.00001**	**100.00%**

### Compute Resources and Training Time

We counted the computing resources (GPU memory) and time required by all the test models in the three experiments during the training, as well as the time required to complete the registration after training. For a fair comparison, all learning-based algorithms are trained under the conditions of batch size = 32 and num points = 1,024. Since ICP and FGR are not training-based models, they do not participate in the comparison of computing resources and training time. As shown in Table 4, our DeepMatch's demand for computing resources is much lower than other learning-based models. DeepMatch can achieve convergence by using fewer epochs, and the training time of each epoch is also the shortest. In terms of overall training time, DeepMatch is at least two orders of magnitude lower than other similarly performing learning-based algorithms, such as RGM. Because of its simple network structure and one-shot registration process, DeepMatch also outperforms other algorithms in terms of registration speed, taking about 0.029 s to complete.

**Table 4:** The computational resources (GPU memories) and time required for training.

**method**	**GPU**	**Time (train)**	**Time (reg)**
ICP	–	–	0.998s
FGR	–	–	0.279s
RPM-Net IDAM	68268M	62h 04m	0.528s
	8697M	5h 10m	0.275s
DeepGMR RGM	3240M	3h 18m	0.499s
	56883M	7h 39m	0.471s
DeepMatch	**2998M**	**34m**	**0.027s**

## Conclusion

For the point cloud registration task, we propose to use DeepMatch to extract the per-point feature from a brand new per-point structure. Based on this per-point feature, DeepMatch achieves state-of-the-art (SOTA) performance on the ModelNet40 dataset and can complete the training and registration with less computing resources and time. We also proved the importance of point cloud size in learning-based algorithms. Through size scaling, the learnability of the point cloud in the point cloud registration task can be improved, so that on unseen categories, our learning-based DeepMatch improves accuracy by two orders of magnitude over RGM, which was the previous best. All in all, we provide you with a way to solve the problem by feature engineering while others are focused on using more complex models, and we will continue to solve the partial to partial point cloud registration problem based on the feature engineering method.

## Data Availability Statement

The original contributions presented in the study are included in the article/supplementary material, further inquiries can be directed to the corresponding author/s.

## Author Contributions

LQ: conceptualization, funding acquisition, and writing. FW: coding and writing. ZG: revising. YS: validation. All authors contributed to the article and approved the submitted version.

## Funding

This study was supported by the Natural Science Foundation of Jiangxi Province (20212BAB202026), the National Key Research and Development Program (Grant No. SQ2020YFF0403429), Shanghai Municipal Science and Technology Major Project (No. 2021SHZDZX0103), Shanghai Engineering Research Center of AI and Robotics, Fudan University, China, and the Engineering Research Center of AI and Robotics, Ministry of Education, China.

## Conflict of Interest

The authors declare that the research was conducted in the absence of any commercial or financial relationships that could be construed as a potential conflict of interest.

## Publisher's Note

All claims expressed in this article are solely those of the authors and do not necessarily represent those of their affiliated organizations, or those of the publisher, the editors and the reviewers. Any product that may be evaluated in this article, or claim that may be made by its manufacturer, is not guaranteed or endorsed by the publisher.

## References

[B1] Aoki Y. Goforth H. Srivatsan R. A. Lucey S. (2019). “Pointnetlk: Robust and efficient point cloud registration using pointnet,” in Proceedings of the IEEE/CVF Conference on Computer Vision and Pattern Recognition, 7163–7172.

[B2] Baker S. Matthews I. (2004). Lucas-kanade 20 years on: a unifying framework. Int. J. comput. Vis. 56, 221–255. 10.1023/B:VISI.0000011205.11775.fd

[B3] Besl P. J. McKay N. D. (1992). “Method for registration of 3-D shapes,” in Sensor Fusion IV: Control Paradigms and Data Structures, vol. 1611. (Bellingham, WA: SPIE), 586–606.

[B4] Campbell D. Petersson L. (2016). “Gogma: Globally-optimal gaussian mixture alignment,” in Proceedings of the IEEE Conference on Computer Vision and Pattern Recognition, 5685–5694.

[B5] Campbell D. Petersson L. Kneip L. Li H. Gould S. (2019). “The alignment of the spheres: Globally-optimal spherical mixture alignment for camera pose estimation,” in Proceedings of the IEEE/CVF Conference on Computer Vision and Pattern Recognition, 11796–11806.

[B6] Censi A.. (2008). “An ICP variant using a point-to-line metric,” in 2008 IEEE International Conference on Robotics and Automation. (Piscataway, NJ: IEEE), 19–25.

[B7] Choy C. Dong W. Koltun V. (2020). “Deep global registration,” in Proceedings of the IEEE/CVF Conference on Computer Vision and Pattern Recognition, 2514–2523.

[B8] Deschaud J. E.. (2018). “IMLS-SLAM: Scan-to-model matching based on 3D data,” in 2018 IEEE International Conference on Robotics and Automation (ICRA). (Piscataway, NJ: IEEE), 2480–2485.

[B9] Fitzgibbon A. W.. (2003). Robust registration of 2D and 3D point sets. Image Vis. Comput. 21,1145–1153. 10.1016/j.imavis.2003.09.004

[B10] Fu K. Liu S. Luo X. Wang M. (2021). “Robust point cloud registration framework based on deep graph matching,” in Proceedings of the IEEE/CVF Conference on Computer Vision and Pattern Recognition, 8893–8902. 10.1109/TPAMI.2022.3204713 36067105

[B11] Gojcic Z. Zhou C. Wegner J. D. Wieser A. (2019). “The perfect match: 3d point cloud matching with smoothed densities,” in Proceedings of the IEEE/CVF Conference on Computer Vision and Pattern Recognition, 5545–5554.

[B12] Han L. Xu L. Bobkov D. Steinbach E. Fang L. (2019). Real-time global registration for globally consistent rgb-d slam. IEEE Transac. Robot. 35, 498–508. 10.1109/TRO.2018.2882730

[B13] Jia S. Ding M. Zhang G. Li X. (2016). “Improved normal iterative closest point algorithm with multi-information,” in 2016 IEEE International Conference on Information and Automation (ICIA). (Piscataway, NJ: IEEE), 876–881.

[B14] Li J. Zhang C. Xu Z. Zhou H. Zhang C. (2020b). Iterative distance-aware similarity matrix convolution with mutual-supervised point elimination for efficient point cloud registration,” in *European Conference on Computer Vision* (Cham, Switzerland: Springer, Cham), 378–394

[B15] Li Y. Ma L. Zhong Z. Liu F. Chapman M. A. Cao D.. (2020a). Deep learning for lidar point clouds in autonomous driving: A review. IEEE Transac. Neural Netw. Learn. Syst. 32, 3412–3432. 10.1109/TNNLS.2020.3015992 32822311

[B16] Low K. L.. (2004). Linear Least-Squares Optimization for Point-to-Plane ICP Surface Registration, vol. 4. (Chapel Hill, CA: University of North Carolina), 1–3.

[B17] Lu W. Zhou Y. Wan G. Hou S. Song S. (2019). “L3-net: Towards learning based lidar localization for autonomous driving,” in Proceedings of the IEEE/CVF Conference on Computer Vision and Pattern Recognition, 6389–6398.

[B18] Lucas B. D. Kanade T. (1981). An Iterative Image Registration Technique with an Application to Stereo Vision, vol. 81, 674–679.

[B19] Qi C. R. Su H. Mo K. Guibas L. J. (2017). “Pointnet: Deep learning on point sets for 3d classification and segmentation,” in Proceedings of the IEEE Conference on Computer Vision and Pattern Recognition, 652–660.

[B20] Rusinkiewicz S. Levoy M. (2001). “Efficient variants of the ICP algorithm,” in Proceedings Third International Conference on 3-D Digital Imaging and Modeling (Piscataway, NJ: IEEE), 145–152.

[B21] Rusu R. B. Blodow N. Beetz M. (2009). “Fast point feature histograms (FPFH) for 3D registration,” in 2009 IEEE International Conference on Robotics and Automation (Piscataway, NJ: IEEE), 3212–3217.

[B22] Rusu R. B. Marton Z. C. Blodow N. Beetz M. (2008). “Persistent point feature histograms for 3D point clouds,” in Proceedings of the 10th Int Conf Intel Autonomous Syst (IAS-10), Baden-Baden, Germany, 119–128.

[B23] Sarode V. Li X. Goforth H. Aoki Y. Srivatsan R. A. Lucey S.. (2019). Pcrnet: Point cloud registration network using pointnet encoding. arXiv preprint arXiv:1908, 07906.

[B24] Wan G. Yang X. Cai R. Li H. Zhou Y. Wang H.. (2018). “Robust and precise vehicle localization based on multi-sensor fusion in diverse city scenes,” in 2018 IEEE international conference on robotics and automation (ICRA), 4670–4677.

[B25] Wang Y. Solomon J. M. (2019a). “Deep closest point: learning representations for point cloud registration,” in Proceedings of the IEEE/CVF International Conference on Computer Vision, 3523–3532.

[B26] Wang Y. Solomon J. M. (2019b). Prnet: self-supervised learning for partial-to-partial registration. Adv. Neural Inf. Process. Syst. 32.

[B27] Wang Y. Sun Y. Liu Z. Sarma S. E. Bronstein M. M. Solomon J. M.. (2019). Dynamic graph cnn for learning on point clouds. Acm Transac. Graph. (tog), 38, 1–12. 10.1145/3326362

[B28] Wu Z. Song S. Khosla A. Yu F. Zhang L. Tang X.. (2015). “3d shapenets: A deep representation for volumetric shapes,” in Proceedings of the IEEE Conference on Computer Vision and Pattern Recognition, 1912–1920.

[B29] Yang J. Li H. Campbell D. Jia Y. (2015). Go-ICP: A globally optimal solution to 3D ICP point-set registration. IEEE Transac. Pattern Anal. Mach. Intell. 38, 2241–2254. 10.1109/TPAMI.2015.2513405 26731638

[B30] Yoo H. Choi A. Mun J. H. (2020). Acquisition of point cloud in CT image space to improve accuracy of surface registration: application to neurosurgical navigation system. J. Mech. Sci. Technol. 34, 2667–2677. 10.1007/s12206-020-0540-6

[B31] Yuan W. Eckart B. Kim K. Jampani V. Fox D. Kautz J.. (2020). “Deepgmr: learning latent gaussian mixture models for registration,” in European Conference on Computer Vision (Berlin, Germany: Springer, Cham), 733–750.

[B32] Zeng A. Song S. Nießner M. Fisher M. Xiao J. Funkhouser T. (2017). “3dmatch: Learning the matching of local 3d geometry in range scans,” in CVPR, Vol. 1 (Honolulu, HI), 4.

[B33] Zhou Q. Y. Park J. Koltun V. (2016). “Fast global registration,” in European Conference on Computer Vision (Berlin, Germany: Springer, Cham), 766–782.

